# Barriers and enablers to implementing telehealth consultations in psycho‐oncology

**DOI:** 10.1002/pon.5939

**Published:** 2022-04-27

**Authors:** Zoe Butt, Laura Kirsten, Lisa Beatty, Brian Kelly, Haryana Dhillon, Joanne M. Shaw

**Affiliations:** ^1^ The University of Sydney School of Psychology Psycho‐Oncology Co‐operative Research Group (PoCoG) Sydney New South Wales Australia; ^2^ Nepean Cancer Care Centre Penrith New South Wales Australia; ^3^ Flinders University College of Education Psychology and Social Work Adelaide South Australia Australia; ^4^ University of Newcastle School of Medicine and Public Health Newcastle New South Wales Australia

**Keywords:** barriers, cancer, COVID‐19, implementation, interviews, oncology, psycho‐oncology, psychologist, qualitative, telehealth

## Abstract

**Objective:**

In response to the COVID‐19 pandemic, use of telehealth to deliver care was recommended across the Australian health system. This study aims to explore the barriers and enablers to delivery of psycho‐oncology services via telehealth and attitudes to use of telehealth in psycho‐oncology.

**Methods:**

Twenty‐one psycho‐oncology clinicians participated in semi‐structured telephone interviews. Transcribed interviews were thematically analysed using the framework method.

**Results:**

Three key themes were identified which described the overall experience of delivering psycho‐oncology services via telehealth: (1) Context *Matters‐for whom is telehealth effective, when is it less effective*; (2) *Therapy content and telehealth implementation*; (3) *Recommendations for* Sustainability.

**Conclusions:**

These insights into the barriers and enablers to delivering psycho‐oncology services via telehealth inform future research and clinical practice. While there is support for the continued use of telehealth in psycho‐oncology, there are significant improvements needed to ensure effective implementation and continued benefit.

## 1 BACKGROUND

In response to the global COVID‐19 pandemic, despite relatively few COVID‐19 cases in Australia, in March 2020 the Australian Government announced immediate measures and restrictions to daily activity and rollout of a universal telehealth model. To minimise risk of COVID‐19 infections, cancer services in Australia rapidly implemented telehealth consultations with the aim of reducing the volume of patients attending hospitals. These changes occurred within hours of health directives being issued impacting health professionals, patients, and their families. For people not infected with COVID‐19, telehealth provided access to cancer care without the risk of exposure.^1^

Telehealth is conceptualised as any *telecommunication that facilitates delivery of care to patients when and where they choose to receive it.* ^2^ Prior to COVID‐19 telehealth in Australia has focused on telephone or video‐conference consultations to improve access for those living outside metropolitan centres. Reported benefits include increased access, time and cost efficiency, improved caseload management and greater patient‐centred care.^3^ From a patient perspective, telehealth reportedly enhances provider‐patient communication and engagement.^4^ Prior to the pandemic there were initiatives to establish telehealth infrastructure and government funding to promote uptake in Australia, although for cancer telehealth models involved clinic‐based shared care between metropolitan cancer centres and local healthcare teams.^5^ Within general mental health services, uptake of telehealth has also been limited, with 2% of all psychiatry consultations and less than 1% of Australian psychology sessions delivered using telehealth.^6^ Telehealth use in the US is reported to be similarly low.^7^ Research suggests psychologists report concerns about efficacy, therapeutic alliance, and the digital divide.^8^ Additionally, while a US study found telehealth to be acceptable psychiatrists noted it was more challenging for patients with complex diagnoses, severe symptoms, and poorer social skills.^9^

The efficacy of telehealth interventions in the context of psycho‐oncology has not been systematically evaluated. A review and meta‐analysis conducted prior to the pandemic reported modest improvements in depression, distress, stress, self‐efficacy and quality of life compared to usual care (face‐to‐face), but conflated telehealth and Internet delivered interventions.^10^ Similarly, a review by Cox and colleagues (2017) highlighted that providing survivorship follow up care via phone, online or via email is viewed positively by cancer survivors due to convenience, provide continuity of care, even at a distance and promotes self management.^11^ Other reviews highlight the non‐inferiority of telehealth more broadly to usual care^12^ and a study evaluating a brain‐cancer specific psychological intervention reported videoconference to be feasible and more acceptable to patients and health professionals than telephone.^13^ A review conducted in 2020 of studies conducted prior to the pandemic highlighted provider satisfaction with telehealth studies^14^ and research specifically exploring barriers to delivery of mental health via telehealth have focused on individual provider acceptance and logistical and administrative issues, with lack of training identified as a key barrier to implementation.^15^, ^16^, ^17^ More recently, a review of transition to telemental health during COVID‐19 (*n* = 9) reported barriers related to technological difficulties; safety and confidentiality, impact of change in medium the patient‐provider relationship and a loss of sense of community.^18^ No psycho‐oncology specific studies were included.

For telehealth to achieve its potential as a mode of healthcare, we must understand the barriers identified by clinicians delivering treatment and identify factors associated with successful integration into routine practice. Our aim was to assess these barriers and facilitators within the COVID‐19 context to establish strategies required for the long‐term maintenance of telehealth within everyday psycho‐oncology practice.

## 2 METHOD

### 2.1 Participants

Participants were Australian psycho‐oncology clinicians (psychologists, psychiatrists, social workers) treating people affected by cancer (patients and/or family members).

### 2.2 Procedure

Participants were recruited through email invitations distributed by professional associations including the Psycho‐oncology Co‐operative Group, Clinical Oncology Society of Australia Psycho‐oncology Group, Psychologists in Oncology, Australian Psychological Society and via a snowballing methodology. Invitations included a link to the participant information statement, consent form, and a short online survey. Consent was obtained through an online consent form and re‐confirmed verbally immediately prior to interview. Recruitment used a purposive sampling approach to identify clinicians with relevant experience and continued until data saturation was achieved.

Qualitative semi‐structured telephone interviews were conducted by a trained qualitative and psycho‐oncology researcher (ZB) not known to the participants May ‐ December 2020. Interview questions (Supplementary File 1) focused on delivery of psycho‐oncology, individual experiences of telehealth and perceived barriers and enablers to wider integration of telehealth into psycho‐oncology. Interview questions were iteratively modified using a constant comparative methodology over the course of the study. Interviews continued until no new information was arising and confirmed with three additional interviews. Field notes were used to explore researcher reflexivity and further support the interpretation of data. The study was approved by the University of Sydney Human Ethics Committee (HREC 2020/380).

### 2.3 Data analysis

Interviews were audio recorded and transcribed verbatim. Transcripts were coded in Nvivo 11 (QSR International) and themes developed through the Framework Analysis method.^19^ Two researchers (ZB and JS) independently read five transcripts line by line and developed initial codes. After discussion, a preliminary working framework was developed and transcripts coded and the coding structure refined as required. Similar concepts were grouped into themes, and patterns between themes and subthemes were identified and mapped into a thematic schema. A framework matrix based on the Framework Analysis approach (Supplementary File 2) was developed, allowing for perspectives across disciplines and groups to be compared. The Consolidated Criteria for Reporting Qualitative Research (COREQ) standards for reporting qualitative research guided reporting.^20^

## 3 RESULTS

### 3.1 Participant characteristics

A total of 22 psycho‐oncology health professionals working clinically were interviewed. One participant was excluded post‐interview as they did not directly provide therapy to people with an experience of cancer. Participants were primarily psychologists (57%, *n* = 12) working in the public setting (82%, *n* = 18) with adult cancer patients (90%, *n* = 19). Participants reported using a combination of telephone and video‐conference technology (67%, *n* = 14) to deliver therapy during COVID‐19. Of those with prior telehealth experience (67%, *n* = 14) most had previously only delivered care via telephone (64%, *n* = 9). See Table 1 for details.

**TABLE 1:** Demographic characteristics

	*N* = 21
Age	
Mean (standard deviation)	43.8 (13.7)
Gender	
Female	0
Male	21
Current profession	
Psychiatrist	1
Clinical Psychologist/Psychologist	12
Social worker	7
Counsellor/Psychotherapist	1
Public/Private system	
Public	18
Private system	0
Mix (public and private)	3
Practice setting	
Tertiary referral Centre	9
Regional Cancer Centre	5
Rural Cancer Centre	0
District/Local Hospital	4
Non‐inpatient Cancer Treatment Centre	2
Other (not‐for‐profit organisation)	1
Oncology population	
Paediatric	0
Adolescents and young adults	2
Adult	19
Mix of the above	0

### 3.2 Qualitative findings

**FIGURE 1:**
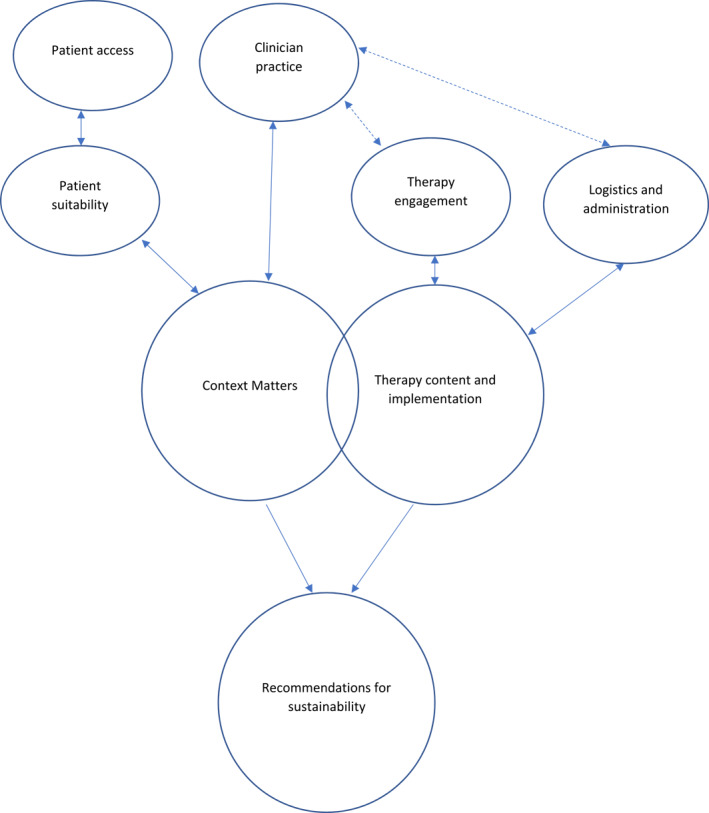
Thematic analysis visual representation

Three themes were identified in the analysis^1^ Context *Matters‐for whom is telehealth effective, when is it less effective*;^2^ *Therapy content and telehealth implementation*;^3^ *Recommendations for* Sustainability. Themes/subthemes are described along with illustrative quotes and a visual representation of themes and proposed inter‐relationships is provided in Figure 1. The use of the term ‘participants’ without qualification, denotes instances where views were expressed to a similar extent by all participants regardless of occupation. The use of a specific role denotes instances where views were expressed only by that group. The use of ‘telehealth’ represents views centred on both phone and video‐conferencing formats, while use of ‘phone’ or ‘video‐conferencing’ where views were expressed only in the context of a specific platform. Additional quotes are detailed in the supplementary materials.

### 3.3 Theme 1: Context Matters‐for whom is telehealth effective, when is it less effective

Multiple contextual factors determined perceived success of telehealth consultations. This included both contexts where telehealth was a useful modality for delivering care (‘where it worked’) and where it was less effective (‘didn't work’).

**1.1 Sometimes it works** Participants reflected on who and when telehealth worked and why it worked in those contexts. Patients geographically isolated, or too sick to travel were often described as the greatest beneficiaries of telehealth.

*I have had patients cancel because they were feeling too tired or they had certain side effect of treatment… I think it [telehealth] actually could overcome some of those other reasons that people choose not to engage…* [P014; Psychologist]

Some participants suggested an existing relationship with patients made telehealth less challenging. Younger patients (<35 years) were also perceived to be more embracing of telehealth presumably because they had greater proficiency with technology.

Telehealth also meant people who would not typically engage with therapy were more willing, due to the sense of anonymity. Others perceived some patients were more comfortable opening up during therapy.

*Psychological services can be quite intimidating for people. So being able to do it at home from a computer, it kind of makes you feel a little bit removed from it, so some people did say that it made them feel a bit more comfortable…* [P013; Psychologist]

Overall, it was perceived that patients generally preferred, and often opted to use, the phone due to its familiarity and ease of use.

*Everyone knows how to use the phone … So that became the main method.* [P023; Psychologist]

Overall, many participants perceived that telehealth was appropriate and helpful under the COVID‐19 restrictions, although it was an option that was “*better than nothing*”.

*The biggest insight I've had is the value of Face‐To‐Face care in clinical psychology. There's so much we do that isn't just verbal. And in order to provide optimal care as a clinical psychologist, face to face is certainly the preference and the gold standard.* [P019; Psychologist]

**1.2** **Sometimes it doesn't** Participants also discussed the many contexts where telehealth is less effective. Some psychologists were reluctant to conduct appointments with people in more complex situations, particularly those with more advanced cancer, or severe mental health concerns and those at risk of self‐harm. These psychologists suggested it was difficult to assess and manage risk without being face‐to‐face with patients.

*People with poor mental health, substance use, complex psychosocial situations, complex family presentations…It works better in the clients whose mental health is not really poor. If they've got mental health issues in the severe range telehealth's not great.* [P004; Psychologist]

Patients too ill to learn a new technology, older patients, a previous poor telehealth experience, and those with lower digital literacy were reported as finding telehealth difficult. A lack of suitable technology was the most common barrier to telehealth use, with patients from low socio‐economic areas lacking access to data required to use video or download exercises.

*Sometimes it's not the element of them not liking it…When they are too unwell they can't be bothered to learn a new software and set it up.* [P016; Psychologist]

It was reported that some patients complained about having too many cancer‐related telehealth appointments. Telehealth was perceived to reduce patient support. Working with linguistically diverse patients was reported to be more challenging over telehealth, particularly when an interpreter was required. In these situations, it was very difficult to build rapport and misinterpretation was frequent. A few participants suggested that telehealth with an interpreter was distressing for patients and suggested where possible face to face sessions were preferable.

*It's very difficult to do phone sessions with interpreters… Not only do you not have the body language, I also don't know the tone that they're using. And sometimes words like anxiety and fear or worry get mixed up…Then sometimes people are talking over the top of each other…So to be honest, I wouldn't recommend doing phone interpreter sessions. It can be quite distressing for the patient and not that helpful.* [P022; Psychologist]

A few psychologists reported privacy concerns, particularly with patients in domestic violence situations, parents with children living at home, and young people living with their parents; all of which contributed to censored discussions. One participant highlighted conducting couples therapy via telehealth, particularly phone as a challenge they had faced.

*My student [provisional psychologist] actually had one situation where she wasn't certain that [the client’s] partner, who was domestically violent, wasn't there…He could have been off on the side while she was saying, “Yes, I'm here on my own.”. She eventually had contact with this person and had a bit of a code worked out so they could make it clear that the other person wasn't there. So that's been a bit of a problem.* [P003; Psychologist]

Alternatively, others suggested that telehealth gave them greater privacy, as prior to COVID‐19 some consultations were routinely delivered in busy clinics or bedside.

*The chemo units are all open so there's not much privacy. Sometimes [patients] want to talk about the nurses or the doctors… They feel more comfortable having a bit of a vent about the whole system when they aren't in the vicinity of the cancer centre*. [P006; Social Worker]

On a personal level, many participants reflected on the high level of fatigue associated with telehealth. Reportedly, telehealth appointments required greater concentration and effort due to the limited non‐verbal cues and trying to read silences, as well as greater focus on building rapport and managing patient distraction. Participants reported the need for breaks between patients or a limit on the number of patients seen per day. Most clinicians indicated they preferred face‐to‐face appointments.

*I found it more emotionally and physically draining…having to try to figure out what the other person, how the other person's reacting and responding without those visual cues*. [p019 Psychologist]

### 3.4 Theme 2: Therapy content and telehealth implementation

Participants described the changes to therapy content and delivery, as well as logistical and administrative considerations associated with telehealth.

**2.1 Therapy engagement** The way in which patients engaged with therapy, as well as how participants provided therapy, changed with the introduction of telehealth. Participants described needing to “*adapt*” their therapeutic practice to fit the telehealth modalities. Some also reported sessions were shorter, supportive counselling rather than therapy sessions.

*It seems to switch from therapy to more of a check‐in service… it's more of a half an hour or even 20 minute check up on how things are going and maybe during a bit of problem solving or focusing on one issue but not doing therapy for longer term issues or processing [P012, Psychologist].*

This sense of ‘checking in’ was heightened by clinicians' difficulty using interactive and experiential exercises. While some found creative ways of adapting these activities, many struggled to effectively include them.

*I think my style now is a lot more reflective, less clinical strategies with people. Maybe just more validation, supportive contact counselling, as opposed to some of those more specific strategies. I can still do mindfulness. That's pretty easy to still do over the phone. ACT is a lot harder because they involve a lot of experiential strategies… And CBT, thought challenging is a bit harder, I'll try to screen share and put it in a word document. It's just a little bit slower, probably not as not as good, maybe, as in person. So yes, I feel like I have definitely altered what clinical skills and interventions I'm doing based on whether I can still do it more verbally rather than in written format [P005; Psychologist]*

Participants also reflected on the loss of silences, visual, and emotional cues. Consequently, many perceived difficulties with building and maintaining a strong therapeutic alliance. One psychologist noted: “*I think in terms of developing trust and rapport, that lack of contact is really detrimental*” [P004]. This was particularly difficult for patients accessing only phone sessions. Similarly, some participants reported the loss of visual cues and the intimate space within sessions made it difficult to discuss more challenging topics associated with cancer, such as death and dying.

*So, it's a little bit harder to talk to someone about death and dying or, you know, whether something that they're doing is harmful or helpful for them, that kind of more pressing, awkward questions can be more difficult to deliver over the phone. Yeah, because you don't know how that other person's going to react or how they'll take it. So it does feel a little bit more surface level.*[P022, Psychologist]

Participants noted the difficulty in developing a complex problem formulation and risk assessment as limited visual information impeded their ability to thoroughly assess a patient, while the distance raised concerns about how to manage high‐risk patients in emergency situations. Being unable to adequately read facial and bodily emotional cues lead to uncertainty of patient's reactions to risk assessments.

*You are really needing to see people's reactions with risk management [and assessment]. There is always the worry of not being able to…contain and manage suicide risk. Being part of a hospital, we have access to an ED department. So we know that we can keep people safe if they are at risk when they come to see us. Whereas by Telehealth and phone that's not possible.* [P019; Psychologist]

Participants noted the ability to see into a patient's home provided them with both benefits and challenges. For some patients, being in their own home allowed them to “*open up and get into a lot of deep therapeutic work*”. But, this also introduced distractions as many initiated therapy sessions while driving, doing household chores or in public settings, prompting a need to re‐schedule appointments to ensure efficacy of interventions.

*I've had sessions with people where I'll be talking to them and then they'll be like, “Oh, sorry, I'm not concentrating. I'm just doing the ironing at the same time.” So people love to try and be really efficient with their time and try and get things all done at once. So I think there's that aspect that's appealing on the surface. But whether or not it's helpful, I'm not sure.* [P022; Psychologist]

Others reported that seeing people's homes provided therapists with more information about the patient. Concurrently, some participants acknowledged the need to maintain boundaries with their patients when they were working remotely.

*I do up a little area…so that there wasn't any identifiable features and things like that…Just for the safety and privacy point of view really.* [P048; Psychologist]

**2.2 Logistics and administrative considerations** Participants discussed practical and logistical challenges that arose. The most common challenge for most participants was technological difficulties using video‐conferencing. Poor Internet connectivity resulted in communication problems and “*disjointed*” therapy sessions. This was reportedly frustrating for both therapist and patient.

*I had concerns around technology failure. Especially with the first appointment with a client. It worries me that they won't want to engage again or they will say, “oh it was too hard”. So, I had some concerns around that. If it was more reliable, maybe I would consider it a little bit more myself* [P024; Counsellor].

### 3.5 Theme 3: Recommendations for sustainability

A range of recommendations were made to increase the suitability and sustainability of telehealth in psycho‐oncology. Firstly, many stressed the need for flexible modes of delivery, based on patient suitability and preference and posited a blended therapy approach may be an appropriate compromise.

*I find meeting them just once helps…I think having that one connection ‐ doesn't have to necessarily be the first one, but somewhere in the trajectory of a relationship, to build that therapeutic relationship…And I think it just helps with the connection that you make with people. [P006; Social Worker]*

Many participants also commented they would benefit from psycho‐oncology specific telehealth guidelines and training, including information on how to adapt therapy approaches to telehealth, patient selection principles and considerations for assessing risk via telehealth.

*I think we probably need more formal guides from Psycho‐oncology in general… Because I'm pretty certain that overtime is going to be differences of opinion on how Telehealth is to be run. So, to have 'this is a recommendation from Psycho‐oncology'* versus *‘this is what I'm using’. It'd be good to have a bit more of like a united front around that. [P016; Psychologist]*

## 4 DISCUSSION

For telehealth to achieve its full potential as a sustainable and effective model of psycho‐oncology care, it is essential to understand the barriers and facilitators to its integration into routine practice. We found there are both benefits and challenges to telehealth. Psycho‐oncology clinicians raised concerns about patient selection and risk management and integrating practical and interactive therapy modalities. In the broader cancer context, balancing the use of telehealth across patient consultations is important to reduce patient and clinician fatigue. For telehealth to be sustainable, these challenges must be addressed to maintain best practice care.

While much discussion of telehealth in the literature has centred on the technology itself,^21^ psycho‐oncology clinicians in this study emphasised therapy engagement as a major challenge to the successful implementation of telehealth in their practice. Importantly, many participants reported difficulty adapting therapy to a telehealth format. Therapies utilising visualisation, physicality and/or interactive exercise, like schema therapy and ACT, were reportedly most difficult to convert to telehealth sessions. Clinicians were cognisant that discussions about existential concerns, death and dying are less distressing face‐to‐face. Participants in our study stressed their eagerness to learn how to overcome this challenge while maintaining high standards of care. Previous telehealth literature has suggested therapists have concerns about treatment efficacy, managing risk and rapport building.^8^ Clinicians in our study highlighted the need for clinical practice guidelines to assist them adapt therapy while maintaining therapeutic effect. They also raised concerns that the therapeutic quality of sessions can be compromised if patients are distracted, and consequently therapeutic effect takes longer to achieve compared to face‐to‐face sessions. This therapeutic effect was reportedly attributed to the screen as a “barrier” for clinicians, reducing visual and non‐verbal cues. This finding is consistent with concerns raised in previous studies about establishing therapeutic alliance.^22^, ^23^

Alternatively, some findings of our study were consistent with the ‘online calming hypothesis’, whereby patients experience the online environment as less threatening than the face‐to‐face setting.^24^ Consistent with previous research reporting the intimacy of being in the patients' home increases therapeutic alliance,^11^ some clinicians reflected on the ability of telehealth to create a safe atmosphere for their patients to engage and discuss more sensitive topics they would not normally raise.

Consistent with the telehealth literature more broadly, patient selection was a key concern identified. There was recognition that for some patients, particularly those geographically isolated and/or too sick to travel, telehealth provided a viable alternative to access therapy. However, risk management associated with more complex cancer and mental health concerns was challenging. Specifically, there were concerns about how to manage individuals assessed as high risk of self‐harm. This finding was similarly discussed in a study assessing telehealth in an outpatient mental health setting where clinicians found managing risk more difficult over telehealth.^9^ These concerns demonstrate a need for more information about managing high‐risk patients, and how to assess whether telehealth is suitable. In the context of AYA psycho‐oncology, Samson‐Daly and Bradford^25^ suggest it is possible to safely conduct telehealth sessions with a range of vulnerabilities, provided risk screening is consistently implemented, and next‐of‐kin contact details are available should acute mental health risk arise. Given this, specific guidance related to risk assessment and management over telehealth is required.

In the context of Australian psycho‐oncology care, which is primarily government‐funded through a universal health system, the digital divide was a major consideration for clinicians. These findings replicate other studies exploring digital disparities.^26^ Access to and familiarity with services delivered via Internet was highlighted as a barrier to wider use of video‐telehealth. To cater to the needs of patients, many services reverted to telephone‐delivered sessions, further limiting clinicians' ability to deliver equitable care. This flexibility reflects other findings highlighting the intent of health professionals in wanting to avoid discriminating against specific groups.^8^ However, as we found, some patients do not have access to the most basic technology, such as the telephone, and they continue to be left behind. Patient preference influenced uptake of psycho‐oncology services, with patients overburdened by the number of telehealth cancer consultations refusing to engage. Other priority populations, for example, culturally and linguistically diverse groups reliant on interpreters to facilitate therapy sessions, were also excluded from the telehealth services. This finding reflects the results of one US study that reported patients with limited English proficiency had lower rates of medical consultations via telehealth compared to English speakers.^27^ Our findings may help to explain this low up‐take of telehealth appointments, as inclusion of interpreters in telehealth sessions is reported to be distressing for some patients.

### 4.1 Clinical implications

For telehealth to be continued to be available while providing the best standard of care possible, a reconceptualization of how clinicians understand and work with telehealth is needed. To overcome barriers to wider implementation, our results demonstrate a need for psycho‐oncology specific guidance on the use of telehealth psycho‐oncology services. This guidance should involve direction around how to adapt therapy approaches to telehealth, recommendations surrounding the groups of patients offered telehealth as well as clinician‐specific strategies for telehealth‐related fatigue.

### 4.2 Study limitations

This study was not without limitations. All clinicians interviewed were female, therefore experiences of male clinicians were not captured. However, this reflects the profession, and we do not expect any gender‐based differences in the experience. Further, the small sample size of self‐selected participants and only one psychiatrist and one counsellor further limits generalisability. This study explored the perspectives of clinicians and the barriers identified may not reflect patient experiences. Lastly, reflecting psycho‐oncology practice in Australia, only three participants were currently working in private sector, further research taking into account the barriers/enablers across the diversity of settings providing psycho‐oncology is required.

## 5 CONCLUSION

Taken together, our findings demonstrate a range of barriers and facilitators to telehealth use in psycho‐oncology. While the introduction of telehealth was largely beneficial and enabled the continued psychological care of cancer patients, there are many challenges that need addressing to improve its sustainability. Psycho‐oncology specific guidelines for clinicians are of increasing importance to support clinicians to provide gold‐standard care while using telehealth platforms.

## CONFLICT OF INTEREST

No conflicts to declare.

## Supporting information

Supplementary Information S1

Supplementary Information S2

## Data Availability

The data is available on request from the authors.
