# Supplementary material for: Barriers and enablers to implementing telehealth consultations in psycho‐oncology
Source: Psychooncology. 2022 Apr 27;31(8):1365–73. doi: 10.1002/pon.5939 (PMC9545227; doi:10.1002/pon.5939)
Supplement: Supplementary file 1 — Supplementary Information S1 [file PON-31-1365-s001.docx]

**Final Interview Schedule with Iterative Changes**

In this interview we will be looking at your general thoughts on use of telehealth in psycho-oncology during the COVID-19 pandemic and the feasibility of implementing telehealth as part of ongoing clinical practice at your current work setting. Telehealth includes telephone or video-conferencing or a combination of both.

Can you tell me a little bit about your experiences with telehealth?

How have you been deciding when to use telephone (or videoconferencing) and when to use face to face approaches?

**Participant perspective of patient experience**

- What do you perceive to be the experiences of patients with telehealth?
  - *What are the barriers and facilitators of a good patient experience with telehealth?*
- Do you think your patients are comfortable using the technology?
- Who do you think telehealth is un/acceptable for?
- Are there any patients missing out/getting lost?
- If patients don’t like telehealth -> why do you think that is? Is it about the technology or is it something about the quality of the sessions that change because of the telehealth?
- Do you perceive there to be any differences in rapport between new and existing patients?
- Have you noticed that there has been a change in referral patterns?
- Have you seen a change in the number of sessions patients are willing to engage in?
- Given that we have been using telehealth for a few months now, do you perceive there are any differences in patient outcomes?

**Clinician Perspective**

- What are your personal views on the use of telehealth?
- Do you feel confident delivering therapy via telehealth?
- What about using telehealth to provide advice to other clinicians? For supervision?
- Have you received any training in the use of the technology?
- Have you received any training in delivery of therapy itself via telehealth
- What would you say are the biggest barriers to telehealth use?
- What would you say are the biggest facilitators for use of telehealth use?
- Has telehealth impacted on your clinical practice? If so, in what ways
- Can you tell me a little bit about delivering telehealth from home and what that has been like for you?
- Has any of the content of your therapy changed to cater for the telehealth platform?
  - If so, what has needed to change in therapy to make telehealth more like face to face appointments?
- If telehealth was available at your hospital *would you continue to recommend telehealth options to your patients? Why, why not?*
- Are there any clients that you think you may not be not accessing since the shift to telehealth?
- How do you think telehealth has impacted your ability to build a therapeutic relationship with your clients?
- Is there anything else you would like to tell me about using telehealth to deliver psycho-oncology?

**Initial Interview Schedule**

In this interview we will be looking at your general thoughts on use of telehealth in psycho-oncology during the COVID-19 pandemic and the feasibility of implementing telehealth as part of ongoing clinical practice at your current work setting. Telehealth includes telephone or video-conferencing or a combination of both.

- Can you tell me a little bit about your experiences with telehealth?
  - *Challenges? Barriers? Facilitators?*
- How have you been deciding when to use telephone (or videoconferencing) and when to use face to face approaches?
- What do you perceive to be the experiences of patients with telehealth?
  - *What are the barriers and facilitators of a good patient experience with telehealth?*
- Do you think your patients are comfortable using the technology?
  - *Any instances where it has worked particularly well? Any where it has not worked well? What were the problems?*
- What are your personal views on the use of telehealth?
- Do you feel confident delivering therapy via telehealth?
- Have you received any training in the use of the technology?
  - *How helpful was it? Changes needed?*
- Have you received any training in delivery of therapy itself via telehealth
- What would you say are the biggest barriers to telehealth use?
- What would you say are the biggest facilitators for use of telehealth use?
- If telehealth was available at your hospital *would you continue to recommend telehealth options to your patients? Why, why not?*
- Is there anything else you would like to tell me about using telehealth to deliver psycho-oncology?
