# Supplementary material for: Barriers and enablers to implementing telehealth consultations in psycho‐oncology
Source: Psychooncology. 2022 Apr 27;31(8):1365–73. doi: 10.1002/pon.5939 (PMC9545227; doi:10.1002/pon.5939)
Supplement: Supplementary file 2 — Supplementary Information S2 [file PON-31-1365-s002.docx]

**Table 1: Stages of Framework Analysis**

| Stage | Description |
| --- | --- |
| 1. Familiarisation with the data | Two researchers (ZB and JS) independently read the transcripts to become familiar with the data |
| 2. Developing a coding framework | Key concepts were used to create a coding framework which could be used to code the data (ZB and JS) |
| 3. Indexing | The thematic framework was systematically applied to code all transcripts and any coding inconsistences were discussed (ZB and JS). The framework was amended as new codes emerged |
| 4. Charting | Coded transcripts were synthesized into a set of thematic matrix charts (ZB) |
| 5. Mapping and Interpretation | The matrix charts allowed for the refinement of the overarching themes and identification of relationships, similarities, and differences within the data (ZB and JS) |
